# Biochemical Characterization, Specificity and Inhibition Studies of HTLV-1, HTLV-2, and HTLV-3 Proteases

**DOI:** 10.3390/life11020127

**Published:** 2021-02-06

**Authors:** Norbert Kassay, János András Mótyán, Krisztina Matúz, Mária Golda, József Tőzsér

**Affiliations:** 1 Department of Biochemistry and Molecular Biology, Faculty of Medicine, University of Debrecen, 4032 Debrecen, Hungary; kassay.norbert@med.unideb.hu (N.K.); matuz.krisztina@med.unideb.hu (K.M.); golda.maria@med.unideb.hu (M.G.); 2 Doctoral School of Molecular Cell and Immune Biology, University of Debrecen, 4032 Debrecen, Hungary

**Keywords:** human T-lymphotropic virus, human T-cell leukemia virus, HTLV-1, HTLV-2, HTLV-3, retroviral protease, protease, HIV protease inhibitor, protease inhibitor

## Abstract

The human T-lymphotropic viruses (HTLVs) are causative agents of severe diseases including adult T-cell leukemia. Similar to human immunodeficiency viruses (HIVs), the viral protease (PR) plays a crucial role in the viral life-cycle via the processing of the viral polyproteins. Thus, it is a potential target of anti-retroviral therapies. In this study, we performed in vitro comparative analysis of human T-cell leukemia virus type 1, 2, and 3 (HTLV-1, -2, and -3) proteases. Amino acid preferences of S4 to S1′ subsites were studied by using a series of synthetic oligopeptide substrates representing the natural and modified cleavage site sequences of the proteases. Biochemical characteristics of the different PRs were also determined, including catalytic efficiencies and dependence of activity on pH, temperature, and ionic strength. We investigated the effects of different HIV-1 PR inhibitors (atazanavir, darunavir, DMP-323, indinavir, ritonavir, and saquinavir) on enzyme activities, and inhibitory potentials of IB-268 and IB-269 inhibitors that were previously designed against HTLV-1 PR. Comparative biochemical analysis of HTLV-1, -2, and -3 PRs may help understand the characteristic similarities and differences between these enzymes in order to estimate the potential of the appearance of drug-resistance against specific HTLV-1 PR inhibitors.

## 1. Introduction

Human T-cell leukemia virus type 2 and type 3 (HTLV-2 and HTLV-3) belong to the delta group of retroviruses, together with HTLV-1, HTLV-4, their simian counterparts (Simian T-cell leukemia viruses, STLVs), and bovine leukemia virus (BLV) [1].

Based on the 2015 report from the European Centre for Disease Prevention and Control [2], at least 5–10 million people are infected worldwide with HTLV-1, while former studies estimated the number of HTLV-1 infected people to be 5–20 million [3]. The data about the worldwide prevalence are limited and are available mainly for HTLV-1 [4]. The estimated numbers imply the decline of worldwide distribution. HTLV-1 is endemic especially in Central-Africa and West-Africa, in Japan, in the Caribbean area, in North-America and South-America, and even in Asia and Europe, while HTLV-2 is most endemic in the United States [5]. As compared to HTLV-1, the number of known HTLV-2-infected people is significantly lower and is estimated to be between 670,000 and 890,000 people [5].

HTLV-1 is associated with adult T-cell leukemia, tropical spastic paraparesis/HTLV-I-associated myelopathy (ATLL, HAM/TSP), or other diseases, such as HTLV-1 associated uveitis or infective dermatitis [6,7]. The epidemiology of HTLV-2 is similar to that of HTLV-1. Interestingly, it can be found in Native American populations [8]. HTLV-2 can cause elevated lymphocyte and thrombocyte counts. Furthermore, it increases cancer mortality [9]. HTLV-3 was isolated only in Cameroon and its association with any diseases has not been published till now, but its similarity to other HTLVs suggests its disease-causing potential.

The life-cycle of the HTLVs is very similar, even though, in the case of HTLV-3, it is barely understood. They prefer different molecules for entry. HTLV-1 requires the presence of heparan sulfate proteoglycans (HSPGs), neuropilin 1 (NRP-1), and glucose transporter type 1 (GLUT-1) on the surface of activated CD4+ T-cells. HTLV-2 requires NRP-1 and GLUT-1 on CD8+ T-cells, while HTLV-3 also targets CD4+ and CD8+ T-cells and its specific receptors are not known [10]. HTLV viruses also infect B-cells, fibroblasts, and macrophages [11,12,13]. There are also functional and structural differences in their accessory/regulatory proteins like Tax, Rex, or HBZ/APH [14,15,16,17,18,19,20].

**Figure 1:**
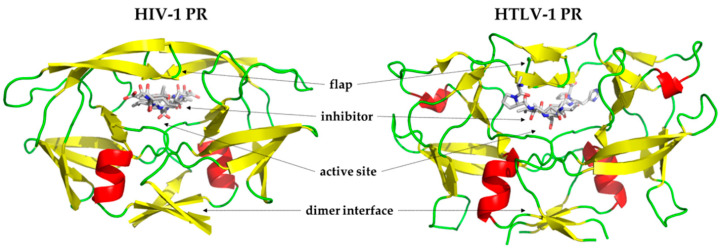
Overall structures of human immunodeficiency virus (HIV-1) and human T-cell leukemia virus type 1 (HTLV-1) viral proteases (PRs). Structures of HIV-1 PR (PDB ID: 5HVP) [23] and HTLV-1 PR (PDB ID: 3LIY) [24] are represented, the inhibitors are bound to the active sites, and the functionally important regions are shown by arrows.

It is common in all retroviruses that the viral protease (PR) has an essential role in the viral life-cycle by processing the Gag and Gag-Pol polyproteins by limited proteolysis, which results in the release of functional proteins, such as matrix (MA), capsid (CA), nucleocapsid (NC), reverse transcriptase (RT), and integrase (IN). The retroviral PRs, including those of HTLVs, are active as homodimers. The dimerization is the prerequisite for enzyme activity [21,22]. They share high structural similarity and use the same catalytic mechanism. The active site contains highly conserved aspartate residues within the consensus D-T/S-G-A active site motif. The catalytic aspartates form a dyad in the homodimeric enzyme. The flap residues are also involved in the formation of substrate binding sites and cover the active site in their closed conformation, wrapping around the ligand (Figure 1).

Currently, there is no standard ATLL therapy, but usually chemotherapy combined with interferon-alpha/zidovudine is used. There are also some potential molecules, which are used in the therapy or are in clinical trials, especially purine analogs, histone deacetylase inhibitors, or monoclonal antibodies [25]. Only some anti-HIV agents including the inhibitors of the nucleoside/nucleotide reverse transcriptase and the protease (PR) are considered to be potential anti-HTLV drugs [26].

The inhibitors that have been developed to target Human immunodeficiency virus type 1 (HIV-1) PR were also considered to be potentially applicable in the treatment of HTLV-1 infection. While HIV-1 protease inhibitors (PIs) are effective inhibitors of HIV-1 PR [27], they are not effective against HTLV-1 PR [28]. Despite the limited potentials of HIV-1 PIs against HTLV-1 PR, the effects of these inhibitors on HTLV-2 and HTLV-3 PRs are still understudied. Our research group previously examined the substrate specificity of HTLV-1 PR using substrate analog series and studied the inhibitory effects of some HIV-1 PIs and two reduced peptide bond-containing HTLV-1 inhibitors (IB-268 and IB-269). The IB-268 and IB-269 HTLV-1 cleavage site analogs and indinavir were found to be relatively good inhibitors of the wild-type HTLV-1 PR [28]. The unsuccessful inhibition by most of the HIV-1 PIs was predicted to be caused by different inter-monomeric interactions of Met37 residue of HTLV-1 PR as compared to Asp30 located in the corresponding position in HIV-1 PR. Thus, new compounds may be designed by targeting this residue [29,30]. Either peptidic or non-peptidic inhibitors may be candidate PIs [31,32,33], but the identification of potent molecules against HTLV PRs is still on demand.

Our previous studies showed that the oligopeptides representing natural cleavage sites of retroviral proteases are effective substrates for HTLV-1 PR, but HTLV-1 PR shows substantially narrower specificity compared to that of HIV-1 PR [28]. In this study, our aim was to test whether the natural cleavage sites of HTLV PRs and other retroviral PRs are effective substrates for HTLV-2 and HTLV-3 PRs in order to compare our results with those of previous studies [28,34,35,36]. We were also curious about differences in enzyme activity, and our aim was to explore how the amino acid differences of substrate binding sites influence the substrate specificity. For this purpose, we used series of P4 to P1′ modified HTLV-1 capsid/nucleocapsid substrates.

## 2. Materials and Methods

All materials were purchased from Sigma-Aldrich (St. Louis, MI, USA). Otherwise, it is indicated.

### 2.1. Expression of HTLV-1, HTLV-2, and HTLV-3 Proteases

The coding sequence of stabilized (C2A) HTLV-1 PR cloned into the pET11a expression plasmid was prepared previously [34]. The sequence of HTLV-2 PR—codon-optimized for bacterial expression—was ordered from GenScript (Genscript Biotech, NJ, USA). The coding sequence of HTLV-3 PR (codon optimized form of Pyl43 strain, Uniprot: Q4U0X6) was cloned from pCR2.1-TOPO plasmid into the pET11a expression vector using NdeI and BamHI restriction endonucleases. It was checked by sequencing.

The expression plasmids were transformed into BL21(DE3) *E. coli* cells (New England Biolabs, Ipswich, MA, USA) using heat-shock at 42 °C for 90 s. The transformed cells were cultured in Luria-Bertani (LB) medium supplemented with ampicillin and grown at 37 °C while shaking until the optical density measured at 600 nm wavelength reached a value between 0.6–0.8. The protein expression was induced by the addition of 1 mM isopropyl β-D-1-thiogalactopyranoside (IPTG), which was followed by incubation at 37 °C for 3 h while continuously shaking.

### 2.2. Purification of HTLV-1, HTLV-2, and HTLV-3 Proteases

After protein expression, the cells were harvested by centrifuging the suspensions at 4000× *g* (Sorvall Lynx 4000, Thermo Fisher Scientific, Waltham, MA, USA) for 20 min at 4 °C. Cell pellets were lysed in buffer A (50 mM Tris-HCl, 1 mM dithiothreitol (DTT), 1 mM ethylenediaminetetraacetic acid (EDTA), pH 8.2). The suspended cells were disrupted by sonication (Branson Sonifier 450, Emerson Electric, MI, USA), which was followed by centrifugation at 25,000× *g* for 20 min at 4 °C (Sorvall Lynx 4000, Thermo Fisher Scientific, Waltham, MA, USA) to prepare a total cell lysate (step 1). To isolate the proteins from inclusion bodies, the cell pellets containing HTLV PRs were first dissolved in buffer B (50 mM Tris-HCl, 1 mM DTT, 1 mM EDTA, 1 v/v% Triton X-100, pH 8.2) (step 2). The latter step was not applied previously in the purification of HTLV-1 PR [28]. After a repeated centrifugation (step 3), the pellets were dissolved in buffer C (50 mM Tris-HCl, 1 mM DTT, 1 mM EDTA, 1 v/v% Triton X-100, 1 M urea, pH 8.2) (step 4). Finally, the solubilized pellets were diluted with buffer D (50 mM Tris-HCl, 7.5 M guanidine-HCl, 5 mM DTT, 5 mM EDTA, pH 8.2) (step 5). The presence of proteins was followed by SDS-PAGE (using 14% or 16% polyacrylamide gels).

After solubilization, the proteins were purified by a reversed-phase high performance liquid chromatography (RP-HPLC)-based method. The purification was performed by Äkta Purifier instrument (Amersham Pharmacia Biotech, Uppsala, Sweden) using the POROS R2 column (Thermo Fisher Scientific, Waltham, MA, USA). For separation, an increasing water/acetonitrile gradient (0–100%) was used in the presence of 0.05% trifluoroacetic acid (TFA) at a 1-mL/min flow rate. The purities of the eluted fractions were determined by SDS-PAGE (using 14% or 16% polyacrylamide gels). The fractions of highest purity (>90%) were applied in downstream steps.

Folding of the enzymes were ensured by dialysis overnight at 4 °C against buffer E (20 mM piperazine-N,N′-bis(2-ethanesulfonic acid) (PIPES), 1 mM EDTA, 100 mM NaCl, 10 v/v% glycerol, 0.5 v/v% NP-40, 5 mM DTT, pH 7.0).

### 2.3. Synthetic Oligopeptides and Inhibitors

The oligopeptides representing the naturally occurring matrix/capsid (MA/CA), capsid/nucleocapsid (CA/NC), trans-frame protein/protease (TF1/PR), and PR/p1 cleavage sites of HTLV-1, -2, and -3 PRs were used as substrates. The oligopeptide substrates representing the natural sequences of HTLV PRs were ordered from BioBasic, while the oligopeptide substrates representing naturally occurring cleavage sites of various viruses [28,37], or the wild-type, the shortened, and the P4, P3, P2, P1, or P1′ variants of HTLV-1 MA/CA cleavage site (KTKVL*VVQPK) were in-house stocks [38]. All peptides were dissolved in distilled water.

The IB-268 (KTKVL-r-VVQPK) and IB-269 (APQVL-r-PVMHP) reduced peptide bond (-r-)-containing inhibitors, which were synthesized by Dr. Ivo Blaha (Ferring Leciva, Prague, Czech Republic). The DMP-323, which is a tight-binding inhibitor of HIV-1 PR [39], and other HIV-1 PIs used in antiretroviral therapy (atazanavir, darunavir, indinavir, ritonavir, and saquinavir) [40] were in-house stocks, and were dissolved in DMSO.

### 2.4. Protease Activity Assays

For kinetic measurements, we applied 10 µL 2× incubation buffer (0.5 M K_3_PO_4_, 4 M NaCl, 10 v/v% glycerol, 10 mM DTT, pH 5.6), 5 µL of HTLV-1, HTLV-2, or HTLV-3 PR (final concentration of the active enzyme was in 0.1–34.4 nM), and 0.5–5 µL oligopeptide substrate representing HTLV PR natural cleavage sites (0.05–1.10 mM final concentration). The final volume of the reaction mixture was set to 20 µL with distilled water if it was necessary. The reactions were initiated by the addition of the enzyme, were incubated at 37 °C for 0.5–4 h, and stopped by the addition of 180 µL 1 v/v% TFA. For substrates representing natural cleavage sites of retroviruses other than HTLV-1, -2, and -3 PRs (HIV-1, equine infectious anemia virus (EIAV), Rous sarcoma virus (RSV), mouse mammary tumor virus (MMTV), Mason-Pfizer monkey virus (MPMV), and murine leukemia virus (MuLV) and BLV) in a longer incubation time (24 h) was also applied. The cleavage products were separated by using a Nova-Pack C18 RP-HPLC column (3.9 mm × 150 mm, Waters Associates Inc, Milford, MA, USA) on HPLC (Merck-Hitachi LaChrom). Water-acetonitrile gradient (0–100%) was used for separation in the presence of 0.05% TFA at 2 mL/min flow rate. The kinetic parameters were determined by fitting the data—obtained at less than 20% substrate hydrolysis—to the Michaelis–Menten equation. The Gaussian equation was used to determine pH optimum and exponential growth equation for determination of optimal NaCl concentration, while linear regression was applied to plot the dependence of enzyme activity on temperature. Prism8 software (GraphPad Software Inc., San Diego, CA, USA) was used for evaluation. The k_cat_/K_M_ catalytic constants were calculated based on the active enzyme concentration, which was determined by active site titration using the method described previously [41].

### 2.5. Determination of PH, Temperature, and Ionic Strength Optimum

To determine the dependence of enzyme activity on ionic strength, pH, and temperature, the enzyme reactions were performed in 2× META buffer (100 mM MES, 200 mM Tris-base, 100 mM sodium acetate). The reaction mixtures contained 10 µL buffer, 5 µL enzyme, and 5 µL substrate. The cleavage reactions were initiated by the addition of the enzyme, and incubated and analyzed as described above in the Section 2.4. The oligopeptides representing HTLV-1 PR/P1, HTLV-2 PR/P1, and HTLV-3 TF1/PR cleavage sites were used as substrates for HTLV-1, HTLV-2, and HTLV-3 PRs, respectively. To determine dependence of activity on NaCl concentration, the final concentration of NaCl ranged from 0 to 2 M. The pH optimum was determined using buffers with a different pH (in 4.5–8 range), while the optimum temperature was determined by measuring activity at different temperatures (20–40 °C).

### 2.6. Determination of Amino Acid Preferences

To compare the amino acid preferences of HTLV-2, and HTLV-3 PRs to those of HTLV-1 PR, the oligopeptides representing the wild-type and the P4, P3, P2, P1, and P1′ variants of HTLV-1 CA/NC cleavage site (KTKVL*VVQPK) were applied as substrates. To study whether S5 and S4 sites contribute to substrate recognition, shortened variants representing P4-P5′ and P3-P5′ residues of the same HTLV-1 CA/NC natural cleavage site were also used. The enzyme reactions were performed as described above in the protease activity assays section. The reaction mixtures contained 10 µL 2× incubation buffer, 5 µL HTLV-1, HTLV-2, or HTLV-3 PR (the final concentration of the active enzyme was 0.1–34.4 nM), and 5 µL of substrate (0.4–0.5 mM final concentration), in a 20-µL final volume. The reactions were initiated by the addition of the enzyme, followed by incubation at 37 °C for 0.5–4 h, prior to HPLC-based separation of 180 µL of 1 v/v% TFA added to the mixtures. The relative activities were determined in the case of all substrates. The activity measured on the wild-type KTKVL*VVQPK substrate was considered to be 100% in the case of each enzyme.

### 2.7. Inhibition Studies

The reaction mixtures were prepared to contain 4.8 µL of substrate (in 0.40–0.43 mM final concentration), 0.2 µL of inhibitor, 5 µL of the enzyme, and 10 µL of a 2× incubation buffer. The substrates representing HTLV-2 and HTLV-3 MA/CA cleavage sites were used for HTLV-2 and HTLV-3 PRs, respectively. In the case of HIV-1 PIs, we applied 100 µM inhibitor stocks, while 0.5–100 µM stocks of IB-268 and IB-269 were used for screening. The inhibitors were dissolved in DMSO. Therefore, 0.2 µL of DMSO was added to the control samples. The IB-269 inhibitor (0–125 nM final concentration range) was applied to determine the amount of the active enzyme.

### 2.8. Studies on Auto-Processing

The coding sequences of HTLV-2 and HTLV-3 PRs containing an eight residue-long N-terminal linker region (corresponding to P8-P1 residues of TF1/PR natural cleavage site at the N-terminus of the viral protease) were cloned into pMALc2x vectors, which enabled expression of the proteins fused to an N-terminal maltose-binding protein (MBP) tag. The cloning procedure included overlap extension PCR, digestion by EcoRI and BamHI restriction endonucleases, and ligation. It was followed by site-directed mutagenesis of HTLV-2 PR (L37D, L37N, L57G, A59I, and F67Q) and HTLV-3 PR (I37D, I37N, L57G, A59I, and F67Q) coding sequences using a QuikChange II mutagenesis kit (Agilent, Santa Clara, CA, USA), according to the manufacturer’s instructions. All applied primers are shown in Table 1. The wild-type constructs and the introduced mutations were verified by sequencing (Genomic Medicine and Bioinformatics Core Facility at University of Debrecen).

The expression constructs were transformed into BL21 (DE3) *E. coli* cells using heat-shock at 42 °C. The recombinant HTLV-2 and HTLV-3 PRs were expressed fused to an N-terminal MBP fusion tag (MBP-HTLV-2 and HTLV-3). The protein expression was induced by the addition of 1 mM IPTG, and then the cells were lysed by sonication in buffer A. The cell lysates were loaded onto 16% polyacrylamide gel. After SDS-PAGE, proteins were transferred onto a nitrocellulose membrane (100 V, 1 h) and the proteins were detected by a Western blot based on the protocol described previously [42]. Anti-MBP monoclonal antibody (E8030S, 1:4000) (New England Biolabs, Ipswich, MA, USA) was used as primary, and an anti-rabbit horseradish peroxidase (HRP)-conjugate (BioRad, Hercules, CA, USA) (170–6515, 1:10,000) as a secondary antibody.

## 3. Results

### 3.1. Expression and Purification of HTLV Proteases

**Figure 2:**
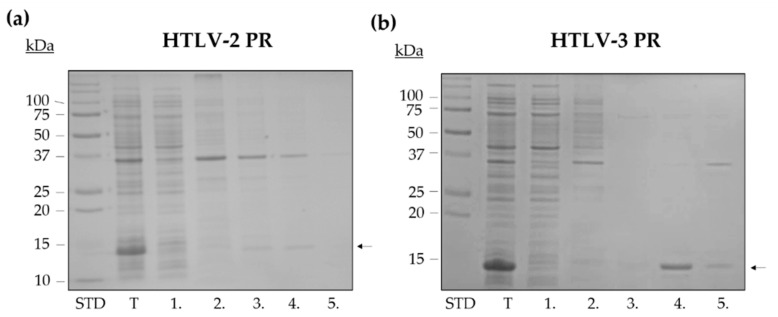
Isolation of HTLV-2 PR (**a**) and HTLV-3 PR (**b**) from inclusion bodies. STD abbreviation indicates the molecular weight standard. The following samples were analyzed by SDS-PAGE: the total cell lysate (T), the supernatant (1), and the pellet dissolved in buffer B (2) after centrifugation of the total cell lysate, the supernatant (3), and the pellet dissolved in buffer C (4) after a repeated centrifugation, and the pellet further diluted with buffer D (5). Arrows show the proteases in the representative gel images.

HTLV PRs were expressed in BL21 (DE3) *E. coli* cells, and each enzyme was purified from inclusion bodies. The extraction of the proteins from the inclusion bodies was the first phase of a purification procedure. The same conditions were applied for each enzyme. The solubilization and purification of HTLV PRs was performed based on a slight modification of the protocol described previously for HTLV-1 PR [28]. We introduced an additional washing step in solubilization (see the details in the Materials and Methods section). The untagged proteins were solubilized using multiple buffer environments and repeated centrifugation steps, which are shown in Figure 2 for HTLV-2 and HTLV-3 PRs.

**Figure 3:**
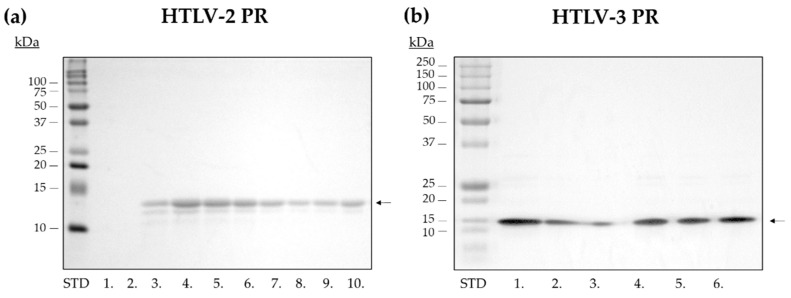
Purification of untagged HTLV-2 and HTLV-3 PRs. Representative gel images show purified fractions of (**a**) HTLV-2 PR (13.8 kDa) and (**b**) HTLV-3 PR (13.3 kDa). The molecular weight standard is indicated as STD, while the collected fractions are numbered (1–8). Fraction 8 (**a**) and fraction 4 (**b**)—having >90% purity—were used for protease assays.

After solubilization, the proteins were purified by an RP-HPLC method. The eluted fractions were collected and the purity of fractions was assessed by SDS-PAGE (Figure 3). The ~37 kDa bands that were present in the samples after solubilization (Figure 2) were predicted to be non-specific contaminants, which were successfully eliminated by downstream chromatographic separation and were not present in the purified fractions of the PRs (Figure 3). The fractions of highest purity (>95%) were dialyzed against buffer E to enable proper protein folding and were used in downstream experiments.

### 3.2. Determination of Optimal Reaction Conditions

First, we tested the effects of different reaction conditions on enzyme activity and determined the dependence of HTLV-2 and HTLV-3 PR activities on temperature, pH, and ionic strength. Later, the optimal conditions—that were found previously to be optimal for HTLV-1 PR [28,43]—were applied in enzymatic reactions.

**Figure 4:**
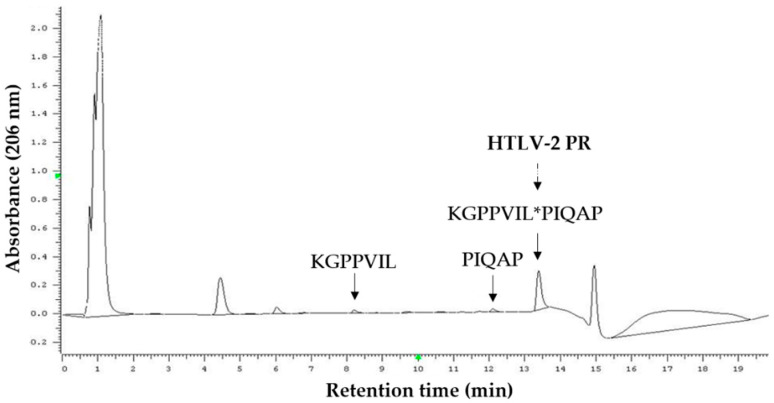
Cleavage of HTLV-1 PR/P1 oligopeptide substrate (KGPPVIL*PIQAP) by HTLV-2 PR. Arrows show peaks of substrate and cleavage products in the representative chromatogram. The substrate and product sequences are also shown. The dashed arrow shows a cleavage position, which is indicated by an asterisk. The cleavage position was determined based on the molecular weights of the substrate and cleavage products determined experimentally by matrix-assisted laser desorption/ionization-time of flight mass spectrometry (MALDI-TOF MS).

For cleavage reactions, the substrates and cleavage products were separated by an RP-HPLC-based method, which is a representative chromatogram shown in Figure 4. For activity measurements, we applied oligopeptide substrates representing natural cleavage sites of HTLV-1, HTLV-2, and HTLV-3 PRs, respectively.

**Figure 5:**
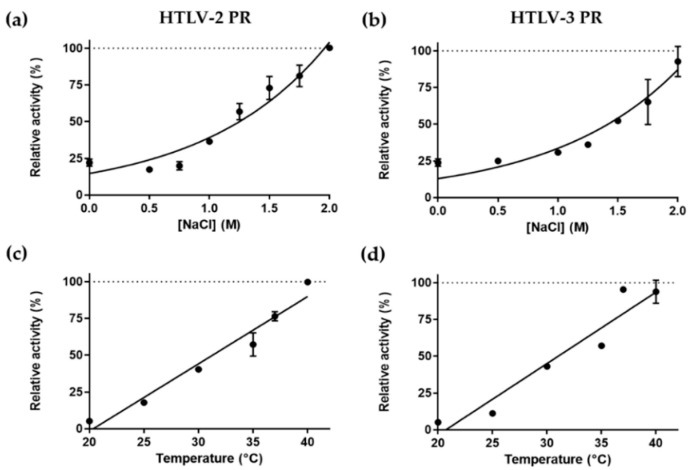
The effect of NaCl concentration (**a**,**b**) and temperature (**c**,**d**) on the activities of HTLV-2 and HTLV-3 PRs. The highest activity was considered to be 100% in each case. Error bars represent SD (n = 2).

Both HTLV-2 and HTLV-3 PRs showed elevated activity at increasing temperatures, the highest activities were measured at ~40 °C, and >50% activity was measured only at a temperature ≥35 °C (Figure 5c,d). In agreement with this, in our previous proteinase assays, we found that 37 °C is sufficient for HTLV-1 PR [28,41], for BLV PR, [43], and for other retroviral proteases as well, including HIV-1 and HIV-2 PR, EIAV PR, MMTV PR, and human foamy virus (HFV) PR [44].

The activities of both HTLV-2 and HTLV-3 PRs were found to be boosted by high ionic strength and showed the highest activity at the highest salt concentration we tested (2 M NaCl) (Figure 5a,b). This is in agreement with the characteristics of HTLV-1 PR, which was also found to show higher catalytic activity at high ion concentration [45]. This dependence of enzyme activity on ionic strength is a common feature of retroviral proteases [46] including HIV-1 PR [47], HFV PR [48], BLV PR [49], and some retroviral-like proteases were also found to share this feature, e.g., the Ty1 retrotransposon PR [50] and the human retroviral-like aspartic protease 1 (ASPRV1) [51].

**Figure 6:**
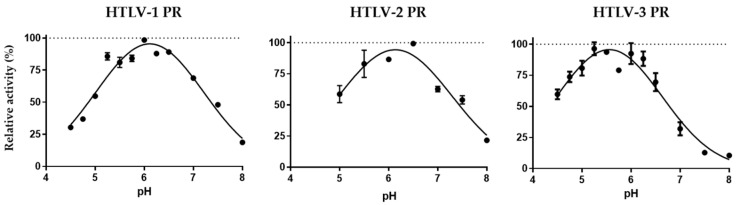
Determination of pH optimum of HTLV proteases. The highest activity was considered to be 100% in each case. Error bars represent SD (n = 2).

The dependence of enzyme activity on pH was studied in a 4.5–8.0 range. We found that pH optimum of HTLV-1 (pH 6.11 ± 0.03) and HTLV-2 (pH 6.14 ± 0.06) PRs are highly similar, while optimal pH for HTLV-3 PR (pH 5.56 ± 0.04) is slightly lower (Figure 6). Our results imply that the optimal pH of HTLV-1 PR is higher than it was determined previously by Ha et al. (pH 5.2–5.3) [52]. The optimal pH determined for HTLV PRs is similar to the slightly acidic pH optimum of retroviral and retroviral-like proteases, including the HIV-1 PR (pH 4.0–6.0 range) [47], the BLV PR (pH 4.0–6.5 range) [49], the HFV PR (pH 6.6) [48], and ASPRV1 (pH 6.27 ± 0.02) [51].

### 3.3. Comparison of Catalytic Efficiencies

We performed activity measurements to determine catalytic efficiencies of HTLV PRs, using oligopeptides representing natural HTLV cleavage site sequences (MA/CA, CA/NC, TF1/PR, or PR/P1) (Table 2). The HTLV-1 PR/P1 site was referred previously as PR/P3 [34] or PR/Px [28] as well. In some cases, longer incubation time (even 4 h) was applied, but this caused no enzyme inactivation or decrease of enzyme activity (Figure S1).

Of the tested substrates, the highest k_cat_/K_M_ values were determined previously for HTLV-1 PR on substrates representing HTLV-1 cleavage sites [34]. Here, we obtained a lower preference for HTLV-2 and HTLV-3 cleavage sites. All oligopeptide substrates were cleaved with the exception of HTLV-3 CA/NC that was not cleaved by HTLV-1 PR. This peptide was an inefficient substrate of HTLV-2 and HTLV-3 PRs as well. For HTLV-1 PR, the lowest k_cat_/K_M_ was obtained by an HTLV-1 PR TF1/PR substrate. Accordingly, we observed the lowest catalytic efficiency with this substrate for HTLV-2 and HTLV-3 PRs among the tested HTLV-1 cleavage sites.

All tested substrates were processed by HTLV-2 PR, the highest catalytic efficiency was obtained on HTLV-2 TF1/PR and PR/P1 substrates. The HTLV-1 and HTLV-2 CA/NC cleavage site sequences differ only in the P5′ residue, which is Lys in the case of HTLV-1 and Arg in HTLV-2. Therefore, only HTLV-1 CA/NC substrate was applied while HTLV-2 CA/NC was omitted from the analysis.

HTLV-3 PR also cleaved all the tested oligopeptides, but, interestingly, the highest k_cat_/K_M_ was obtained for the HTLV-1 PR/P1 cleavage site, which was the most preferred substrate of HTLV-1 PR as well.

We decided to perform activity measurements with substrates representing HIV-1, EIAV, RSV, MMTV, MPMV, BLV, and MuLV PR cleavage sites. However, HTLV-1 PR was found previously to cleave some of these substrates [28]. We observed no processing by HTLV-2 and HTLV-3 PRs in any case (Table 3), which indicates a broader specificity for HTLV-1 PR as compared to HTLV-2 and HTLV-3 PRs. Previously, some of the tested peptides were found to inhibit either HTLV-1 or HIV-1 PR at higher than 0.1 mM concentrations [28]. Therefore, it is important to note that some of the substrates may have an inhibitory effect on HTLV-2 and HTLV-3 PRs as well.

### 3.4. Determination of Amino Acid Preferences

**Figure 7:**
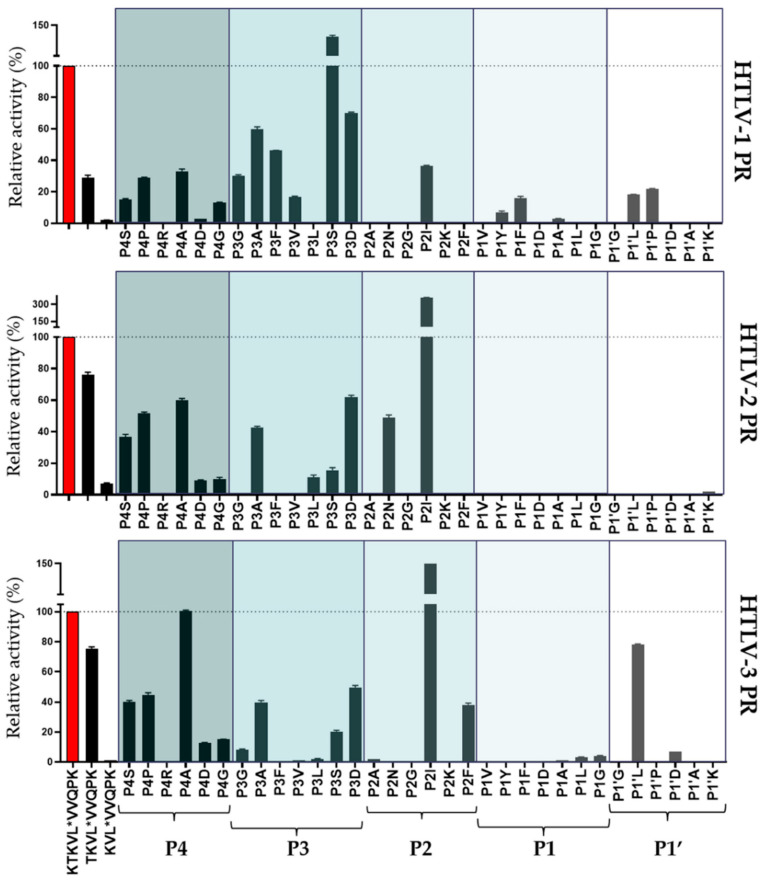
Comparison of the specificity of HTLV-1, HTLV-2, and HTLV-3 PRs using shortened and substituted analogs of HTLV-1 CA/NC oligopeptide substrate. Activity measured on the wild-type KTKVL*VVQPK substrate was considered to be 100%. Only >1% relative activities are plotted. Error bars represent SD (n = 2).

For the comparative analysis of amino acid preferences, activity measurements were performed to determine relative activities of HTLV PRs using series of oligopeptide substrates. Each substrate was modified from the wild-type HTLV-1 CA/NC (KTKVL*VVQPK) substrate by shortening its length (P5-P5′, P4-P5′, and P3-P5′) or by modifying single positions (P4, P3, P2, P1, or P1′) (Figure 7).

We found that each HTLV PR showed lower activity on shortened substrates as compared to the wild-type (KTKVL*VVQPK). The shortest substrates (P3-P5′) were not processed or only negligible hydrolysis was detected (Figure 7). In agreement with the lower catalytic constants obtained previously for HTLV-1 PR [38], the relative activities determined in this study also imply lower preference of each HTLV PR for the shortened substrates and the significant contribution of S4 and S5 sites to substrate recognition in HTLV-2 and HTLV-3 PRs as well.

The mutations at the P4 position were well tolerated by each HTLV PR. This is in agreement with the relatively larger hydrophobic S4 cavity of HTLV-1 PR [28,44,53]. The screening of P4 variants resulted in similar amino acid preferences for HTLV PRs. The lowest relative activity was observed for P4-Gly and P4-Asp mutants, while none of the enzymes showed processing of the P4-Arg variant (Figure 7).

Based on the relative activities obtained for the tested P3 variants, various residues can be accommodated at the S3 subsite. The amino acid preferences were similar, but only HTLV-1 PR accepted P3-Phe and P3-Val mutants as substrates, and showed higher preference for a P3-Ser residue as compared to P3-Asp. In the case of HTLV-1 PR, the P3-Ser variant was the only substrate for which higher relative activity was observed than for the wild-type.

The P2-Ile mutant was found to be the most preferred substrate of the tested P2 variants, and the relative activity obtained for HTLV-2 and HTLV-3 PRs was higher as compared to the wild-type substrate (Figure 7). Similar to our previous screening of P2 mutants [38], we also found that binding of Ile to the S2 site is preferable and P2-Asn and P2-Lys residues are not accepted by HTLV-1 PR. P2-Asn variant was a relatively good substrate of HTLV-2 PR, even though similarities of substrate binding site compositions (see below) did not imply this.

Tyr and Phe were tolerated in the P1 position only by HTLV-1 PR. These variants were found previously to be good substrates of this enzyme [38]. HTLV-3 was the only enzyme which did not cleave P1-Ala mutant and processed P1-Leu and P1-Gly variants with low turnover. HTLV-2-PR did not tolerate the substitutions at the P1 site. Only some of the variants were cleaved but with lower efficiency (<1%) as compared to the wild-type substrate. Only low activity values were obtained for HTLV-3 PR from assays performed with P1-Ala, P1-Leu, and P1-Gly substituted substrates (Figure 7).

In agreement with our previous results [38], variants containing Asp, Gly, or Lys in the P1′ position were not cleaved by HTLV-1 PR, and were inefficient substrates for HTLV-2 and HTLV-3 PRs as well. The P1′-substituted oligopeptides were not effective substrates for HTLV-2 and HTLV-3 PRs with the exception of a P1′-Leu substituted substrate, which was efficiently cleaved by HTLV-3 PR (Figure 7).

### 3.5. Inhibition of HTLV Proteases

**Figure 8:**
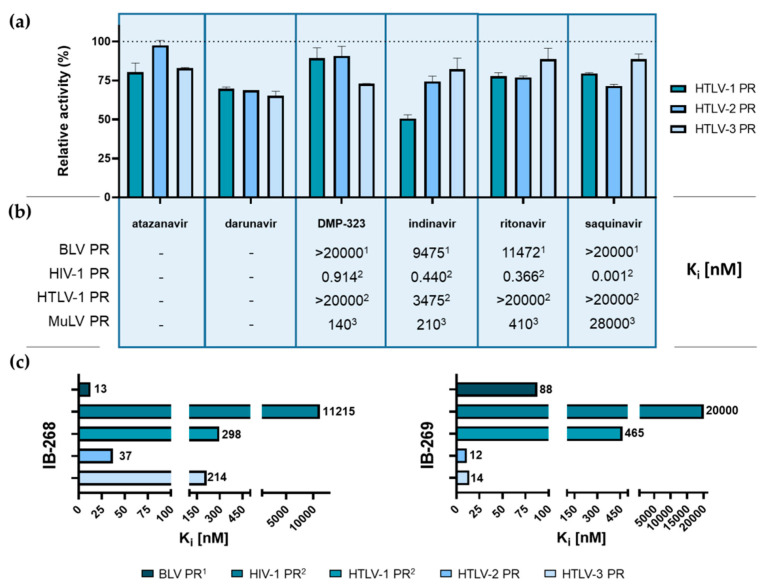
Inhibition of HTLV-1, HTLV-2, and HTLV-3 PRs by different inhibitors. (**a**) Comparison of relative activities determined in the presence of HIV-1 PIs applied in a 1-µM final concentration. For control measurements, reaction mixtures contained no inhibitor. Activity determined in the presence of DMSO was considered to be 100%. Error bars represent SD (n = 2). (**b**) For comparison of relative efficacies, Ki values available for BLV ^1^ [37], MuLV ^3^ [39], HIV-1 ^2^ [41], and HTLV-1 ^2^ [41] PRs were obtained from the literature. The referred data are comparable as each was obtained from Edans/Dabcyl fluorescent oligopeptide-based measurements. Literature values are shown in the table only for inhibitors where data are available for all four assays. (**c**) Comparison of Ki values determined for IB-268 and IB-269 inhibitors in the case of BLV ^1^ [37], HIV-1 ^2^ [41], HTLV-1 ^2^ [41], HTLV-2, and HTLV-3 PRs.

We tested the inhibitory potentials of different PIs including approved therapeutic HIV-1 PIs (atazanavir, darunavir, indinavir, ritonavir, and saquinavir) and DMP-323, which is a tight-binding inhibitor of HIV-1 PR. All tested PIs showed only moderate inhibitory effect against HTLV-2 and HTLV-3 PRs, and we observed <50% loss of activity even when using the inhibitors in a 1-µM final concentration (Figure 8a). The observations are in agreement with literature data. The lowest inhibitory constants were measured for HIV-1 PR, while these inhibitors were found to be less potent against BLV, HTLV-1, and MuLV PRs (Figure 8b).

Ritonavir has already been tested as a potential inhibitor against HTLV-1 PR. This HIV-1 PI was found to have anti-leukemic activity against ATLL cells ex vivo [54], but the inhibitory effect was considered to be a consequence of a ritonavir-dependent inhibition of NF-κB transcriptional activation in ATLL cells rather than impaired activity of HTLV-1 PR [55]. Accordingly, we found that ritonavir is not an effective inhibitor of HTLV-1 PR, and did not inhibit either HTLV-2 or HTLV-3 PR. The anti-tumoral effect of ritonavir via inhibition of NF-κB activation renders it the best HIV-1 PI with therapeutic potential in treating an HTLV-1 infection [10].

We found that darunavir and indinavir showed the highest inhibition of the studied inhibitors in the case of each protease, but the most remarkable inhibition was observed for indinavir, which caused a ~50% decrease of HTLV-1 PR. In all other cases, more moderate inhibition was observed, which is in agreement with the results of previous studies revealing that the HIV-1 PIs are not effective against HTLV-1 PR [56].

Inhibition studies were also performed to study the inhibitory potential of IB-268 (KTKVL-r-VVQPK) and IB-269 (APQVL-r-PVMHP) HTLV-1 PIs. The K*i* value for HTLV-1 PR has already been determined previously for IB-268 (298 nM) and for IB-269 (465 nM) [28]. These inhibitors were applied previously for the inhibition of HIV-1 PR, and both IB-268 and IB-269 were found to be much less effective as compared to the approved therapeutic HIV-1 PIs [41]. We repeated these experiments in our system for HTLV-2 and HTLV-3 PRs. The K*i* values obtained for HTLV-2 and HTLV-3 PRs were lower as compared to those determined for HTLV-1 PR (Figure 8c). IB-268 was found to be a more effective inhibitor for HTLV-2 while ~6-fold higher K_i_ was determined for HTLV-3 PR. IB-269 was found to be more effective against both HTLV-2 and HTLV-3 PRs as compared to IB-268. The obtained K*i* values were highly similar (12 and 14 nM, respectively).

### 3.6. Comparison of Protease Sequences and Substrate Binding Site Compositions

**Figure 9:**
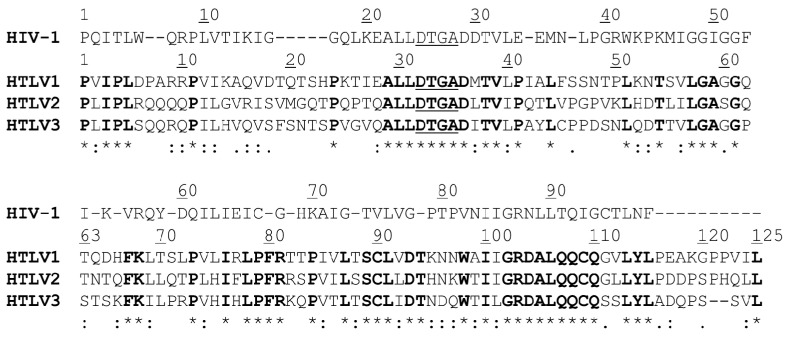
Sequence alignment of HIV-1 and HTLV PRs. Structure-based alignment of HIV-1 and HTLV-1 PR sequences was performed previously [57]. Consensus active site motif residues are underlined. Sequence numbering is shown for HIV-1 and HTLV PRs. Residue similarity (., :) and identity (*) is shown only for the alignment of HTLV PR sequences. The residues that are identical in HTLV PRs are bold.

The proteases of HTLV-1, HTLV-2, and HTLV-3 viruses show no high sequence identity (Figure 9). HTLV-1 shares 50% and 49% sequence identity with HTLV-2 and HTLV-3 PRs, while latter ones show 57% sequence identity. Despite the low sequence identity between these enzymes, the substrate binding residues were found to be more conserved and show 82%, 56%, 76%, and 59% identity for S1, S2, S3, and S4 subsites, respectively. The compositions of S4-S1 substrate-binding cavities have already been determined for HTLV-1 PR [44,53], and the binding site-forming residues of HTLV-2 and HTLV-3 PRs (Table 4) were identified based on sequence alignment (Figure 9).

### 3.7. Studies on Auto-Processing

**Figure 10:**
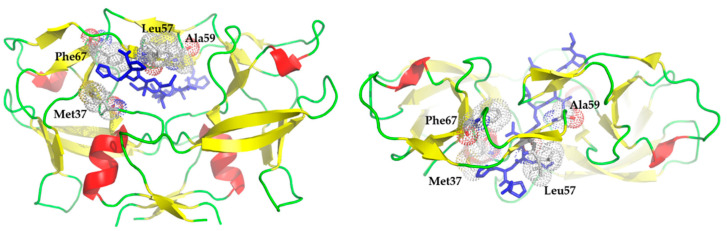
Structure of HTLV-1 protease. Structure of HTLV-1 PR complexed with a statine-containing peptide inhibitor is represented in front and top views (PDB ID: 3LIY) [24]. The inhibitor is shown by a blue color, while surfaces of the mutated residues in the 37th, 57th, 59th, and 67th positions are shown by dots.

We prepared mutant HTLV-2 and HTLV-3 PRs, which contained mutations that were introduced previously to HTLV-1 PR to study its self-processing capability [28]. The rationale behind the former mutation design was to prepare mutant HTLV-1 PRs that contain HIV-1-like residues in the flap region (57th and 59th) or at the active site (37th and 67th). The herein studied enzymes also contained the same mutations in the corresponding positions (Figure 10). All modified residue constitutes a part of substrate binding sites (Table 4). The 37th residue is involved in the formation of S2 and S4 sites, while the 57th residue is part of the S3 site as well. The 59th and 67th residues were in the S1/S2 and S4 sites, respectively. Leu57 is conserved among the studied HTLV PRs and considered to be part of the flap of HTLV-2 and HTLV-3 PRs as well. It is known that Leu in this position (G48L mutation) contribute to resistance of HIV-1 PR against indinavir and saquinavir [58]. Furthermore, L57G mutant HTLV-1 PR was found to be an inefficient enzyme if the activity was measured with the HTLV-1 CA/NC substrate [28].

In the case of HIV-1 and HIV-2 PRs, the I50 residue—corresponding to A59 of HTLV PRs—is suggested to have a role in flap mobility. A59I mutation in HTLV-1 PR was found previously to cause undetectable activity [28].

F67 is considered to be part of the S4 subsite and its change to Gln was also not tolerated by HTLV-1 PR [28].

**Figure 11:**
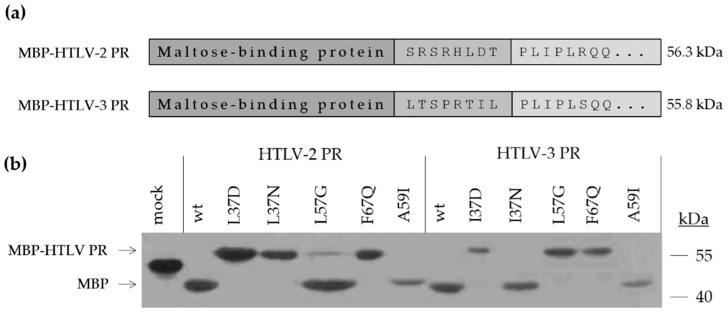
Schematic representation of MBP-HTLV-2 and three PR fusion proteins. (**a**) Fusion proteins contain an N-terminal MBP tag (dark grey), which is connected to the protease domain (light grey) by an eight residue-long short linker representing a natural sequence of the polyprotein prior to the protease (grey). (**b**) Ability of wild-type and mutant enzymes for autoproteolysis was studied by a Western blot. We applied an empty pMalc2x vector to express the MBP protein only (mock). The recombinant protein translated from the empty vector (in the case of mock control) has higher molecular weight as compared to the MBP released from the MBP-HTLV PR fusion proteins by proteolysis, due to the presence of an extension in its C-terminus.

HTLV-2 and HTLV-3 PRs were expressed as fusion proteins containing an N-terminal MBP tag. There was an 8-residue-long linker sequence between the tag and the enzyme in both cases in which the sequence represented the N-terminal flanking sequences of the proteases (P8-P1 residues). This linker enabled the proteases to process themselves from the fusion protein. This sequence was SRSRHLDT in the HTLV-2 PR, and LTSPRTIL in the HTLV-3 PR (Figure 11a). The self-processing of HTLV-2 and HTLV-3 PRs were determined by following the product formation by a Western blot (Figure 11b). The observed effects of mutations were compared to the results obtained previously for HTLV-1 PR mutants (Table 5).

The results showed a similar effect of mutations on autoproteolysis in the case of each enzyme. Differences were observed in the case of mutations at the 37th and 57th positions (Table 5). Mutation of the 37th residue to Asn prevented autoproteolysis of HTLV-1 and HTLV-2 PRs, while I37N mutant HTLV-3 PR retained its ability for self-processing (Figure 11b) (Table 5).

It was found previously that HTLV-1 PR bearing the individual L57G mutation was a very inefficient enzyme and did not hydrolyze the CA/NC cleavage site peptide (KTKVL*VVQPK). Therefore, its ability for self-processing was not studied [28]. In agreement with this, in our experiments, the L57G mutation prevented self-cleavage of HTLV-3 PR, but the same single point mutation did not abolish the ability of HTLV-2 PR for autoproteolysis (Figure 11b).

## 4. Discussion

In this study, we aimed the characterization of HTLV-2 and HTLV-3 PRs and the comparison of their main biochemical features and specificities with those of HTLV-1 PR.

The enzymes were expressed in BL21(DE3) *E. coli* cells and, after their extraction from inclusion bodies, they were purified by an RP-HPLC-based method. First, we investigated the effects of different reaction conditions on enzyme activity to determine optimal conditions. We found that similarly to HTLV-1 PR and numerous retroviral PRs, the optimal temperature for HTLV-2 and HTLV-3 PRs is close to 37 °C, and their activity is boosted by high ionic strength (even by 2 M NaCl) (Figure 5). Optimal pH determined for HTLV-1 and HTLV-2 PRs was highly similar (pH 6.1) while HTLV-3 PRs showed a lower pH optimum (pH 5.6) (Figure 6), but each value was in agreement with the slightly acidic pH optimum of most retroviral and retroviral-like proteases. The optimum pH of an aspartic PR could be anywhere from a fairly acidic (pepsin) to a neutral (renin) pH. For retroviral PRs, the evolved pH optimum may reflect the entry pathway. In receptor-mediated endocytosis, the pH optimum could be the pH of the endosomes (acidic) while, in direct fusion, it could be more like the intracellular pH. Additionally, the high salt concentration was found previously to boost HIV-1 PR activity by increasing conformational stability, and the unfolding rate constant and enzyme stability were also found to be the lowest at the optimal pH [59].

For the comparison of the substrate specificities of HTLV PRs, we used series of oligopeptides representing naturally occurring cleavage sites of HTLV PRs (Table 2) and various retroviruses (Table 3), and series of P4-P1′-modified substrates were also screened (Figure 7). In our experiments, we applied oligopeptide substrates and used an HPLC-detection assay, and the main reaction conditions (temperature, pH, ionic strength) also correspond to those of our former studies. Thus, our results obtained for HTLV-2 and HTLV-3 PRs are comparable with the values determined previously for HTLV-1 PR [28,34]. The peptides representing HIV-1, EIAV, RSV, MMTV, MPMV, BLV, and MuLV PR cleavage sites were found previously to be more or less good substrates of HIV-1 PR, and some of them were cleaved by HTLV-1 PR as well [28]. In our experiment, none of these peptides were hydrolyzed by HTLV-2 and HTLV-3 PRs (Table 3). Screening of P4-P1′-modified substrates also revealed somewhat similar amino acid preferences for each HTLV PR, but specificities of HTLV-2 and HTLV-3 PRs were more similar, which was expected based on their higher sequence identity compared to HTLV-1 PR. While 17 out of the studied 32 peptides (53%) was cleavable by HTLV-1 and HTLV-3 proteases with a variable efficiency. Only 11 (34%) was cleavable by the HTLV-2 enzyme (relative activities obtained for P4-P1′-modified substrates are shown in Figure 7). It has already been reviewed that some retroviral PRs (e.g., HIV-1, MMLV, BLV PRs) have much broader specificity than HTLV-1 PR [60], but our results imply a narrower specificity for HTLV-2 and HTLV-3 PRs than for HTLV-1 PR. The higher stringency is in agreement with their evolutionary relationships, as HTLV-2 and HTLV-3 PRs are more similar to each other than to HTLV-1 PR [61].

In contrast to the differences in their specificity, our results imply that the size of a substrate-binding site is similar in each HTLV PR. As compared to the decapeptide, we observed lower cleavage efficiency for the N-terminally shortened nonapeptides and octapeptides, lacking P5 and P5-P4 residues, respectively (Figure 7). This reveals that the outer (S5 and S4) sites also contribute to substrate binding in HTLV-1, HTLV-2, and HTLV-3 PRs. HTLV-1 PR was previously found to have a substrate groove, which enables interactions with P12-P5 (and P5′-P12′) substrate residues in the enzyme surface [62]. Our previous results have already provided evidence for the contribution of the S5 site to the substrate recognition of HTLV-1 PR [38], and our study revealed that this site has a significant role in substrate binding of HTLV-2 and HTLV-3 PRs as well. However, future studies may be necessary to analyze interactions of extended substrates (e.g., P12-P12′) with the enzyme surface and characterize a substrate groove in HTLV PRs in detail.

The inhibition profiling of the three HTLV PRs revealed that those HIV-1 PIs, which were found previously to be inefficient against HTLV-1 PR [28,34,41] have no significant inhibitory effects against HTLV-2 and HTLV-3 PRs. Accordingly, HTLV PRs are not direct molecular targets of atazanavir, darunavir, indinavir, ritonavir, and saquinavir, indicating that they have no promising therapeutic potential in the treatment of HTLV infections. IB-268 and IB-269 inhibitors were found to inhibit HTLV-2 and HTLV-3 PRs more efficiently as compared to HTLV-1 and HIV-1 PRs. IB-269 was the most potent inhibitor of HTLV-2 and HTLV-3 PRs. K*i* was found to be between 12 and 14 nM, respectively. This was similar to the inhibition constant determined previously for BLV PR [37]. Ritonavir is the only known HIV PI, which can be applied in the treatment of HTLV infection since it can inhibit NF-κB activation, but it acts on alternative target(s) rather than the viral protease [54]. Thus, the identification of potent therapeutic PIs against HTLV PRs is still in demand [26].

To assess the tolerance of HTLV-2 and HTLV-3 PRs toward mutations likely appear in the development of resistance towards PIs. Protease mutants were also designed to contain HIV-1-like residues in the active site, at 37th, 57th, 59th, and 67th positions, which correspond to the 30th, 48th, 50th, and 58th positions in HIV-1 PR. Being part of the ligand-binding sites, they are prone to mutations in drug resistance. The effect of mutations on the enzyme folding and activity were studied by investigating self-processing of MBP-fused enzymes containing a natural cleavage site sequence prior to the protease. None of the single mutant HTLV-1 PRs were found to retain their ability for autoproteolysis upon single point mutations (M37D, M37N, and F67Q) [28], but, in contrast with this, we found that the M37N mutant HTLV-3 PR was still able to self-process itself. These results imply that the flap residues (at least M37) may contribute differentially to the stabilization and folding of HTLV PRs. The structural background of this need to be revealed by future studies.

Retroviruses can replicate by two fundamentally different mechanisms. The replication can occur by forming exogenous virions, and infection of target cells, like in the case of HIV, but also in the integrated DNA form, by forcing the infected cells to divide, as seen in the case of HTLV-1. The first method involves the action of two error-prone enzymes, known as RNA polymerase II and RT, resulting in accumulation of mutations that may contribute to a much better mutation tolerance of the viruses. This enables the quick development of resistance against any kind of drugs. Our previous studies suggested that HTLV-1 PR is much more sensitive toward binding site mutations as compared to HIV-1 PR [28]. Although the sequence of HTLV-2 and HTLV-3 PRs is as different from that of HTLV-1 as seen in comparison of HIV-1 and HIV-2 PRs, their similar replication strategy (replicate predominantly in the DNA form) provided a much more rigid substrate specificity and more or less similar mutation intolerance for these enzymes, while HIV-1 underwent more rapid evolution and its PR became highly tolerant for mutations under the selective pressure.

In summary, our result provides important information about biochemical characteristics of HTLV-2 and HTLV-3 PRs, which may aid design of specific inhibitors, as identification of an effective treatment to control HTLV-1 infection and to treat HTLV-1-related diseases is still an ongoing challenge.

## Figures and Tables

**Table 1:** The oligonucleotide primers used for cloning of human T-lymphotropic viruses (HTLV-2) and HTLV-3 viral proteases (PRs) and for site-directed mutagenesis. All primers were ordered from Sigma-Aldrich. FWD and REV indicate forward and reverse primers, respectively.

Name	FWD/REV	Primer Sequence
HTLV-2 PR N-terminal linker	FWD	5′-AGGATTTCAGAATTCCCCGACCAGGACATCTCAATACTTCCGCTGATTCCGCTGCGTCAAC-3′
	REV	5′-CTAGAGGATCCTTACAGCAGTTGATGCGGTGACGGGTCGTCCGGC-3′
HTLV-3 PR N-terminal linker	FWD	5′-AGGATTTCAGAATTCCTTACATCTCCACGTACAATTCTTCCCCTCATACCCTTGTCCCAACAAAG-3′
	FWD	5′-GCGGATCCTTAGAGAACACTTGAGGGTTG-3′
HTLV-2 PR L37D	FWD	5′-GGCGCGGACGATACGGTCATT-3′
	REV	5′-AATGACCGTATCGTCCGCGCC-3′
HTLV-2 PR L37N	FWD	5′-GGCGCGGACAACACGGTCATT-3′
	REV	5′-AATGACCGTGTTGTCCGCGCC-3′
HTLV-2 PR L57G	FWD	5′-ACGCTGATCGGCGGCGCCAGT-3′
	REV	5′-ACTGGCGCCGCCGATCAGCGT-3′
HTLV-2 PR A59I	FWD	5′-ATCCTGGGCATTAGTGGTCAG-3′
	REV	5′-CTGACCACTAATGCCCAGGAT-3′
HTLV-2 PR F67Q	FWD	5′-AACACGCAACAGAAACTGCTG-3′
	REV	5′-CAGCAGTTTCTGTTGCGTGTT-3′
HTLV-3 PR I37D	FWD	5′-GGGGCGGACGATACTGTTCTC-3′
	REV	5′-GAGAACAGTATCGTCCGCCCC-3′
HTLV-3 PR I37N	FWD	5′-GGGGCGGACAACACTGTTCTC-3′
	REV	5′-GAGAACAGTGTTGTCCGCCCC-3′
HTLV-3 PR L57G	FWD	5′-ACCACTGTCGGCGGCGCAGGC-3′
	REV	5′-GCCTGCGCCGCCGACAGTGGT-3′
HTLV-3 PR A59I	FWD	5′-GTCTTAGGCATTGGCGGGCCA-3′
	REV	5′-TGGCCCGCCAATGCCTAAGAC-3′
HTLV-3 PR F67Q	FWD	5′-ACCAGCAAGCAGAAGATCCTG-3′
	REV	5′-AGGATCTTCTGCTTGCTGGT-3′

**Table 2:** Catalytic efficiencies of HTLV PRs on different substrates representing natural cleavage site sequences. Cleavage sites are labelled by asterisks within the sequences. Abbreviations: MA (matrix), CA (capsid), NC (nucleocapsid), and TF1 (trans-frame 1).

		k_cat_/K_M_ [mM^−1^ s^−1^]		
Substrate	Sequence	HTLV-1 PR	HTLV-2 PR	HTLV-3 PR
HTLV-1 MA/CA	APQVL*PVMHP	85.2 ± 26.1 ^1^	5.5 ± 1.0	32.5 ± 6.7
HTLV-1 CA/NC	KTKVL*VVQPK	150.6 ± 15.1 ^1^	67.2 ± 33.4	30.1 ± 15.4
HTLV-1 TF1/PR	DPASIL*PVIP	3.8 ± 0.6 ^1^	0.4 ± 0.1	1.2 ± 0.4
HTLV-1 PR/P1	KGPPVIL*PIQAP	288.3 ± 73.6 ^1^	20.6 ± 2.8	238.4 ± 60.1
HTLV-2 MA/CA	TTQCF*PILHP	14.4 ± 4.3	4.3 ± 0.5	37.8 ± 20.2
HTLV-2 TF1/PR	SPRTIL*PLIP	4.9 ± 1.1	132.5 ± 42.5	5.0 ± 1.1
HTLV-2 PR/P1	PHQLL*PIATP	10.7 ± 4.8	92.2 ± 41.4	7.5 ± 2.7
HTLV-3 MA/CA	ASQCL*PILHP	40.9 ± 10.4	2.6 ± 0.4	19.7 ± 4.4
HTLV-3 CA/NC	KNKIL*MIQPK	not cleaved	1.4 ± 0.2	0.8 ± 0.3
HTLV-3 TF1/PR	PRTIL*PVIPL	109.8 ± 29.3	11.8 ± 2.0	11.8 ± 2.0
HTLV-3 PR/P1	PSKVL*PVLAP	7.0 ± 2.6	2.1 ± 0.3	22.6 ± 5.8

^1^ These values have already been reported [34].

**Table 3:** Catalytic efficiencies of HTLV PRs on different substrates representing natural cleavage site sequences of viruses other than HTLV. Cleavage sites are labelled by an asterisk within the sequences. Abbreviations: MA (matrix), CA (capsid), NC (nucleocapsid), TF (transframe), RT (reverse-transcriptase), and IN (integrase).

Substrate	Sequence	k_cat_/K_M_ (mM^−1^ s^−1^)			
		HIV-1 PR ^1^	HTLV-1 PR ^1^	HTLV-2 PR	HTLV-3 PR
HIV-1 MA/CA	VSQNY*PIVQ	45.3	not cleaved	not cleaved	not cleaved
HIV-1 CA/P2	KARVL*AEAMS	90	not cleaved	not cleaved	not cleaved
HIV-1 P2/NC	TATIM*MQRGN	74	not cleaved	not cleaved	not cleaved
HIV-1 NC/P1	ERQAN*FLGKI	1	not cleaved	not cleaved	not cleaved
HIV-1 TF/PR	VSFNF*PQITL	6.9	not cleaved	not cleaved	not cleaved
HIV-1 PR/RT	CTLNF*PISP	24.1	not cleaved	not cleaved	not cleaved
EIAV MA/CA	PSEEY*PIMID	15.2	0.7	not cleaved	not cleaved
EIAV PR/RT	AKLVL*AQLSK	13.4	not cleaved	not cleaved	not cleaved
EIAV RT/RH	KEEIM*LAYQG	18.3	<0.01	not cleaved	not cleaved
RSV P2B/P10	PPYVG*SGLYP	not cleaved	not cleaved	not cleaved	not cleaved
RSV P10/CA	PVVAM*PVVIK	not cleaved	0.1	not cleaved	not cleaved
RSV CA/P3	IAAAM*SSAIQ	not cleaved	not cleaved	not cleaved	not cleaved
RSV P3/NC	IQPLIM*AVVNR	318	>100	not cleaved	not cleaved
RSV NC/PR	PPAVS*LAMTM	0.13	not cleaved	not cleaved	not cleaved
RSV PR’/RT	RATVL*TVALH	1.9	0.3	not cleaved	not cleaved
RSV RT/IN	TFQAY*PLREA	0.18	not cleaved	not cleaved	not cleaved
MMTV MA/PP21	SDLVL*LSAEARR	6.9	not cleaved	not cleaved	not cleaved
MMTV PP21/P3	DSKAF*LADTW	7.5	not cleaved	not cleaved	not cleaved
MMTV P3/P8	DELIL*PVKRK	1.5	2.6	not cleaved	not cleaved
MMTV P8/N	PPVGFAG*AMA	<0.01	not cleaved	not cleaved	not cleaved
MMTV N/CA	LTFTF*PVVFMRR	0.9	0.01	not cleaved	not cleaved
MPMV P12/CA	PKDIF*PVTET	0.2	0.2	not cleaved	not cleaved
BLV MA/CA	PPAIL*PIISE	0.3	164.5	not cleaved	not cleaved
BLV CA/NC	KQPAIL*VHTPG	not cleaved	not cleaved	not cleaved	not cleaved
BLV PR/P13	PPMVG*VLDAP	0.04	0.7	not cleaved	not cleaved
MuLV MA/P12	PRSSLY*PALTP	0.2	not cleaved	not cleaved	not cleaved
MuLV P12/CA	TSQAF*PLRAG	8.7	not cleaved	not cleaved	not cleaved
MuLV NC/PR	TQTSLL*TLDDQ	not cleaved	not cleaved	not cleaved	not cleaved
MuLV RT/IN	TSTLL*IENSS	not cleaved	not cleaved	not cleaved	not cleaved

^1^ Data for HIV-1 and HTLV-1 PRs were retrieved from the literature. The residues that were added to the cleavage site sequences to enhance the solubility of the peptides are underlined [28]. The increase of RSV P3/NC substrate concentration was found previously to cause a decrease in activity of HTLV-1 PR [28]. The residues that were added to the cleavage-site sequences to enhance the solubility of the peptides are underlined.

**Table 4:** Comparison of S4-S1 binding site-forming residues in HIV-1 and HTLV PRs. For HTLV PRs, the residues that are different in the equivalent positions are bold and underlined.

S4				S3				S2				S1			
HIV-1 *	HTLV-1 *	HTLV-2	HTLV-3	HIV-1 *	HTLV-1 *	HTLV-2	HTLV-3	HIV-1 *	HTLV-1 *	HTLV-2	HTLV-3	HIV-1 *	HTLV-1 *	HTLV-2	HTLV-3
D30	**M37**	**L37**	**I37**	R8	**R10**	**Q10**	**Q10**	A28	A35	A35	A35	R8	**R10**	**Q10**	**Q10**
M46	**S55**	**L55**	**T55**	D29	D36	D36	D36	D30	**M37**	**L37**	**I37**	L23	L30	L30	L30
I47	**V56**	**I56**	**V56**	G48	L57	L57	L57	V32	V39	V39	V39	D25	D32	D32	D32
G48	L57	L57	L57					I47	**V56**	**I56**	**V56**	G27	G34	G34	G34
V56	F67	F67	F67					G48	L57	L57	L57	G49	G58	G58	G58
Q58	**L69**	**L69**	**I69**					G49	G58	G58	G58	I50	A59	A59	A59
L76	L91	L91	L91					I50	A59	A59	A59	T80	-	-	-
								L76	L91	L91	L91	P81	-	-	-
								I84	I100	I100	I100	V82	W98	W98	W98
												I84	I100	I100	I100

* Binding site compositions were determined previously for HIV-1 and HTLV-1 PRs [44,53].

**Table 5:** Effects of protease mutations on autoproteolytic activity of HTLV PRs.

Position/New Residue	HTLV PR	Enzyme Form	Self-Processing
-	HTLV-1 *	wild-type	yes
	HTLV-2	wild-type	yes
	HTLV-3	wild-type	yes
37 (D)	HTLV-1 *	M37D	no
	HTLV-2	L37D	no
	HTLV-3	I37D	no
37 (N)	HTLV-1 *	M37N	no
	HTLV-2	L37N	no
	HTLV-3	I37N	yes
57 (G)	HTLV-1 *	L57G	not determined
	HTLV-2	L57G	yes
	HTLV-3	L57G	no
59 (I)	HTLV-1 *	A59I	not determined
	HTLV-2	A59I	yes
	HTLV-3	A59I	yes
67 (Q)	HTLV-1 *	F67Q	no
	HTLV-2	F67Q	no
	HTLV-3	F67Q	no

* HTLV-1 PR mutants were designed and characterized previously [28].

## Data Availability

Data is contained within the article or Supplementary Materials.

## Supplementary Materials

The following are available online at https://www.mdpi.com/2075-1729/11/2/127/s1. Figure S1: Effect of incubation time on the activities of HTLV-1, HTLV-2, and HTLV-3 PRs.
