# Supplementary material for: Biochemical Characterization, Specificity and Inhibition Studies of HTLV-1, HTLV-2, and HTLV-3 Proteases
Source: Life (Basel). 2021 Feb 6;11(2):127. doi: 10.3390/life11020127 (PMC7915765; doi:10.3390/life11020127)

life

MDPI

Supplementary material

Biochemical Characterization, Specificity and Inhibition Studies of HTLV-1, HTLV-2, and HTLV-3 Proteases

Norbert Kassay [1,2,3], János András Mótyán [1,*], Krisztina Matúz [1,2], Mária Golda [1,3] and József Tőzsér [1,*]

1 Department of Biochemistry and Molecular Biology, Faculty of Medicine, University of Debrecen, 4032 Debrecen, Hungary; kassay.norbert@med.unideb.hu (N.K.); matuz.krisztina@med.unideb.hu (K.M.); golda.maria@med.unideb.hu (M.G.)
2 Department of Pharmacology, Faculty of Medicine, University of Debrecen, 4032 Debrecen, Hungary
3 Doctoral School of Molecular Cell and Immune Biology, University of Debrecen, 4032 Debrecen, Hungary
* Correspondence: motyan.janos@med.unideb.hu (J.A.M.); tozser@med.unideb.hu (J.T.); Tel.: (+36)-52/512-900

Figure S1. Effect of incubation time on the activities of HTLV-1, -2 and -3 PRs. Experiments were performed as it is described in *Protease activity assays* section of Materials and Methods. HTLV-3 PR/P1 oligopeptide was used as substrate in each reactions. Error bars represent SD (n=2).

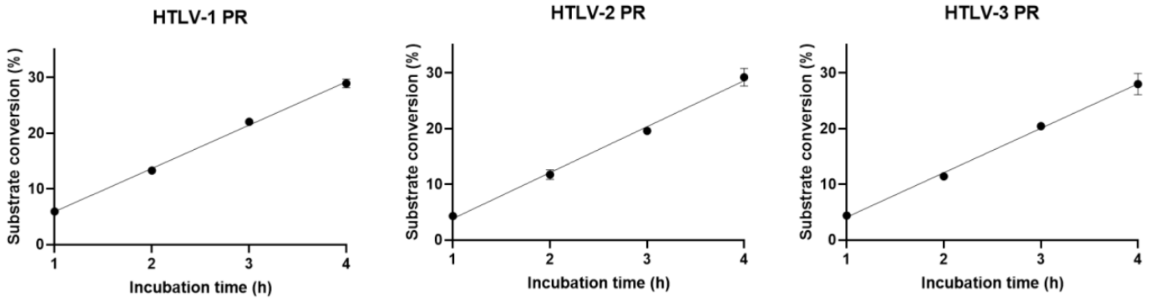

Supplement: Supplementary file 1 [file life-11-00127-s001.pdf]
